# A whole genome-based characterization of *Listeria monocytogenes* strains isolated from invasive infections in Northwest Italy, 2022–2025

**DOI:** 10.3389/fmicb.2026.1867228

**Published:** 2026-06-15

**Authors:** Monica Pitti, Matteo Tavecchia, Clara Tramuta, Angelo Romano, Simona Carrella, Giovanna Previto, Carlotta Polizzi, Teresa Zaccaria, Daniela Manila Bianchi

**Affiliations:** 1 Istituto Zooprofilattico Sperimentale del Piemonte, Liguria e Valle d’Aosta, S.C. Sicurezza Alimentare, Turin, Italy; 2 Centro di Riferimento per la Tipizzazione delle Salmonelle, CeRTiS, Istituto Zooprofilattico Sperimentale del Piemonte Liguria e Valle d’Aosta, Turin, Italy; 3 Laboratorio di Microbiologia e Virologia, Azienda Ospedaliero-Universitaria, Città della Salute e della Scienza di Torino, Turin, Italy

**Keywords:** antibiotic resistance genes, cgMLST, *Listeria monocytogenes*, virulence genes, whole-genome sequencing

## Abstract

**Introduction:**

*Listeria monocytogenes* is a primary foodborne pathogen and the causative agent of human listeriosis and remains one of the most critical foodborne pathogens responsible for severe invasive infections with high hospitalization and mortality rates. According to the European Food Safety Authority (EFSA) and the European Centre for Disease Prevention and Control (ECDC), listeriosis continues to show a stable trend, particularly among elderly and immuno-compromised populations. The integrations of Whole Genome Sequencing (WGS) data into routine surveillance represent a key advancement in food safety monitoring and rapid detection and resolution of outbreaks. Since March 2022, the Regional Reference Centre for Salmonella Typing (CeRTiS) has implemented WGS as a standard typing tool for the public health surveillance of *L. monocytogenes*.

**Methods:**

This study presents WGS-derived data characterizing 135 *L. monocytogenes* strains collected from human cases in Northwest Italy between 2022 and 2025.

**Results:**

High-resolution typing based on cgMLST and phylogenomic analysis identified 36 sequence types grouped into 29 clonal complexes (CCs). The most prevalent were: CC1 (20.7%), CC3 (13.3%), CC155 (10.3%), CC8 (7.4%), and CC6 (5.2%).

**Discussion:**

The predominance of Lineage I complexes, specifically CC1 and CC3, which are traditionally associated with invasive clinical disease indicates the persistent circulation of hypervirulent clones. Furthermore, the detection of ST155, recently linked to multinational clusters reported by the ECDC, highlights the necessity of supranational genomic surveillance in tracking the spread of potential epidemic lineages. This study reinforces the role of genomic epidemiology as a cornerstone of modern One Health strategies for preventing and controlling listeriosis.

## 1 Introduction

*Listeria monocytogenes* is the etiological agent of listeriosis, a severe foodborne infection affecting humans and various vertebrate species (Muchaamba et al., 2022). Invasive listeriosis occurs primarily in people with existing chronic illness, immunocompromised patients, pregnant women, older adults and newborns causing sepsis, meningoencephalitis and abortion (Lee et al., 2018). Healthy adults infected with *L. monocytogenes* may show gastroenteritis with diarrhea and vomiting (Lee et al., 2018). Listeriosis remains associated with the highest hospitalization and mortality rates in Europe. According to the European Food Safety Authority (EFSA) and the European Centre for Disease Prevention and Control (ECDC), listeriosis continues to show a stable or increasing trend, particularly among elderly and immunocompromised populations (Ricci et al., 2018). Recent data from the EFSA/ECDC reported 0.69 cases of listeriosis per 100,000 population in 2024, 3.0% higher than in 2023 (0.67 per 10,000 population), confirming the increase of listeriosis cases per year (European Food Safety Authority [EFSA], and European Centre for Disease Prevention and Control [ECDC]., 2025). The overall trend for listeriosis cases in the period 2020–2024 in Europe showed a significant increase. Recently, a genomic cluster of *L. monocytogenes*, belonging to sequence type (ST) 155, infections has been identified in the European Union and the United Kingdom: the outbreak affected Austria, Belgium, Italy, Germany and the Netherlands, with 17 cases reported and two deaths: the infections mainly involved elderly people (European Centre for Disease Prevention and Control, 2023). In USA it is the third leading cause of death from food-borne infections (0.31 cases per 100,000 people) (Shah et al., 2024)

Transmission of *L. monocytogenes* is primarily foodborne, with ready-to-eat (RTE) products, dairy items, meat products, and fish-derived foods representing the main vehicles of infection (Hofer et al., 2006). Moreover, the ability of this microorganism to survive under refrigeration, tolerate high salt concentrations, and persist in food processing environments makes it a significant challenge for food safety systems (Carpentier and Cerf, 2011; Ricci et al., 2018). Persistent contamination within processing facilities may facilitate the long-term circulation of specific clonal complexes, some of which are strongly associated with invasive human disease (Orsi et al., 2011).

*L. monocytogenes* is classified into thirteen serotypes (1/2a, 1/2b, 1/2c, 3a, 3b, 3c, 4a, 4ab, 4b, 4c, 4d, 4e, and 7) and four major molecular serogroups (IIa, IIb, IIc, and IVb) (EURL Lm, 2021). Of these serovars 1/2a, 1/2b, and 4b are involved in over 95% of human clinical cases (Lee et al., 2018): serovar 1/2a was frequently isolated from contaminated foods, while 4b exhibits the strongest epidemiological association with human listeriosis (Pan et al., 2009). The population structure of *L. monocytogenes* is highly clonal and organized into distinct lineages, sequence types (STs), and clonal complexes (CCs). There are four evolutionary lineages of *L. monocytogenes* (I, II, III and IV): Lineage I includes isolates of serotypes 1/2b, 3b, 4b, 4d, 4e, and 7, Lineage II isolates of serotypes 1/2a, 1/2c, 3a, and 3C, Lineage III serotypes 4b, 1/2a, 4a, and 4c and Lineage IV isolates of serotypes 4a and 4c (Haase et al., 2014). Strains belonging to Lineages I and II are mainly isolated from food, environment and human outbreaks of listeriosis, whereas Lineages III and IV strains are less common and isolated from ruminant animals (Orsi et al., 2011).

The capacity of *L. monocytogenes* to invade host cells depends on several virulence factors, including actin assembly-inducing protein (ActA), internalins, invasion-associated protein (p60), listeriolysin O (LLO), phospholipases, and regulatory system for gene expression of virulence (PrfA) (Parussolo et al., 2021). These virulence factors are encoded by genes that are usually located on genomic and pathogenic islands (LIPI-1to LIPI-4) (Radoshevich and Cossart, 2018). Large-scale genomic studies have demonstrated that a limited number of hypervirulent clones, particularly within Lineage I, are associated with invasive infections: hypervirulent clonal complexes, including CC1, CC4, and CC6, which are frequently linked to bloodstream and central nervous system infections (Maury et al., 2016).

This microorganism has been generally reported susceptible to almost all antibiotics, that have bactericidal activity against gram-positive bacteria (Carpentier and Cerf, 2011; Orsi et al., 2011), but naturally resistant to cephalosporins, fosfomycin and macrolides (Carpentier and Cerf, 2011). In recent years, resistant strains have been reported more frequently among human, food, plant and environment isolates (Orsi et al., 2011, Pan et al., 2009).

A variety of molecular subtyping techniques have been developed to characterize *L. monocytogenes* isolates, including pulse-field gel electrophoresis (PFGE), multilocus variable-number tandem-repeat analysis (MLVA), multilocus sequence typing (MLST), and whole genome sequencing (WGS) (Osek et al., 2022). In recent years, WGS has become the reference method for high-resolution typing, enabling precise strain discrimination, detection of outbreak clusters, and identification of virulence and antimicrobial resistance determinants. The integration of WGS into routine surveillance systems supports a One Health approach, bridging clinical microbiology and food safety monitoring.

Since March 2022, the Regional Reference Centre for Salmonella Typing (Centro di Riferimento per la Tipizzazione delle Salmonelle, CeRTiS) has implemented routine WGS for all *invasive L. monocytogenes* isolates from human in Northwest Italy referred by local laboratories (CeRTiS Clinical Laboratories Group) for public health surveillance.

The aim of this study was to characterize the genomic population structure of invasive *L. monocytogenes* isolates collected between 2022 and 2025. Specifically, we assessed the distribution of serogroups, sequence types (STs), virulence factors, resistance genes and major clonal complexes, to determine the circulating clones in Piedmont and Liguria regions (Northwest Italy), thereby strengthening the public health and food safety framework.

## 2 Materials and methods

### 2.1 Sample collection, *Listeria monocytogenes* isolation and identification

A total of 135 *L. monocytogenes* isolates were collected through laboratory-based passive surveillance in the Piedmont and Liguria regions between March 2022 and March 2025. It should be noted that this surveillance system focuses on isolate characterization; therefore, detailed patient demographic data and clinical metadata were not available for the laboratory. For each suspected clinical episode, only a single representative isolate per patient was included in the study to avoid redundancy. Strains were isolated from blood or cerebrospinal fluid samples of patients presenting invasive listeriosis. Two to three blood culture sets were collected for each suspected episode of bloodstream infection, each consisting of one aerobic and one anaerobic bottle.

Blood culture bottles were incubated in a continuous-monitoring automated system (BACT/ALERT Virtuo, bioMérieux, Marcy l’Étoile, France) and processed according to the manufacturer’s instructions. Bottles were monitored until positivity or until completion of the standard incubation period (5 days). When growth is detected, a Gram stain was performed, and an aliquot was subcultured onto Columbia blood agar (CBA) and MacConkey agar (Mk) plates using an automated specimen processor (WASPLab, Copan, Italy). CBA plates were incubated at 37°C in a CO2 atmosphere, while Mk plates were incubated aerobically at the same temperature overnight (18–24 h). Growth on CBA under CO2 conditions was typically observed earlier and allowed preliminary evaluation of colony morphology and hemolytic activity. Identification of isolated colonies was performed by matrix-assisted laser desorption/ionization time-of-flight mass spectrometry (MALDI-TOF MS) using the Bruker system (Bruker Daltonics GmbH & Co. KG, Bremen, Germany), according to the manufacturer’s instructions.

*L. monocytogenes* isolates were stored at −80°C in appropriate cryopreservation media until further molecular analysis.

### 2.2 Serogrouping

Multiplex PCR was utilized for molecular serogrouping of the isolates (IIa, IIb, IIc, IVa, IVb), targeting the genes *prf*A, *prs*, *lmo0737, lmo1118, orf2819*, and *orf2110* (D’Agostino et al., 2004; Doumith et al., 2004). Genomic DNA was extracted using InstaGene Matrix (BioRad, Hercules, CA, United States) according to the manufacturer’s instructions. The thermal cycling included a pre-warming step at 95°C for 15 min, followed by 35 cycles consisting of denaturation at 95°C for 30 s, annealing at 58°C for 90 s and extension at 72°C for 90 s, concluding with a final extension at 72°C for 10 min. The serogroup assignment was determined based on amplification profiles.

### 2.3 Whole genome sequencing

WGS was used for the molecular characterization of the *L. monocytogenes* strains, enabling the identification of Sequence Types (STs), Clonal Complexes (CCs), core genome MLST (cgMLST) types, and the presence of virulence and antimicrobial resistance genes. DNA extraction was performed using the Extractme Genomic DNA Isolation Kit (Blirt, Gdańsk, Poland) from single colonies grown on Columbia Blood Agar (Biolife, Milan, Italy) for 24 h at 37°C. Before applying the manufacturer’s protocol, a pre-lysis step was included, consisting of an incubation for 30 min at 37°C with 105 μL of lysozyme (10 mg/mL). DNA quantification was carried out using Qubit Fluorometer (Thermo Fisher Scientific, Waltham, MA, United States), and library preparation was conducted using the Illumina DNA Library Prep Kit (Illumina, San Diego, CA, United States) according to the manufacturer’s protocol. The sequencing run was performed on an Illumina MiSeq platform (Illumina, San Diego, CA, United States) employing MiSeq V2 and V3 chemistry to produce 2 × 151 bp paired-end reads.

### 2.4 Bioinformatics analysis

Bioinformatics analysis of the raw data produced was carried out through bioinformatic tools and pipelines available on the GenPat platform (Italian National Reference Centre for WGS of Microbial Pathogens—available online: https://genpat.izs.it/cmdbuild/ui/#custompages/welcomePage, accessed on 25 January 2026). Raw reads were trimmed using Trimmomatic by remove the Illumina adaptors and other Illumina-specific sequences [Illuminaclip set to Nextera (paired-ended)], by removing low-quality residues at the start and the end of the reads (leading:10 and trailing:10), by clipping the reads when the average Q-scores dropped below 20 over a sliding window of four residues (slidingwindow:4:20), and by dropping the reads shorter than 40 bases after processing (minlen:40). The trimmed reads were assembled *de novo* using Unicycler for the bridging mode moderate contig size and misassembly rate (the bridging mode was set to normal) and the contigs below 200 bp in length were excluded (excluded contigs from the FASTA file which were shorter than this length (bp) set to 200).

The assembled genomes were processed using MLST tool that performs Multi Locus Sequence Typing *in silico*, assigning both Sequence Type (ST) and Clonal Complex (CC), scanning contig files against traditional PubMLST typing schemes.

Acquired antimicrobial resistance genes (ARGs) were identified using ABRicate (v1.0.1) with a minimum sequence identity threshold of 85% and a minimum coverage of 60% searching against the databases: argannot (Jul 14 2020), card (Jul 14 2020), resfinder (Jul 14 2020), ncbi (Jul 14 2020), vfdb (Jul 14 2020).

**FIGURE 1:**
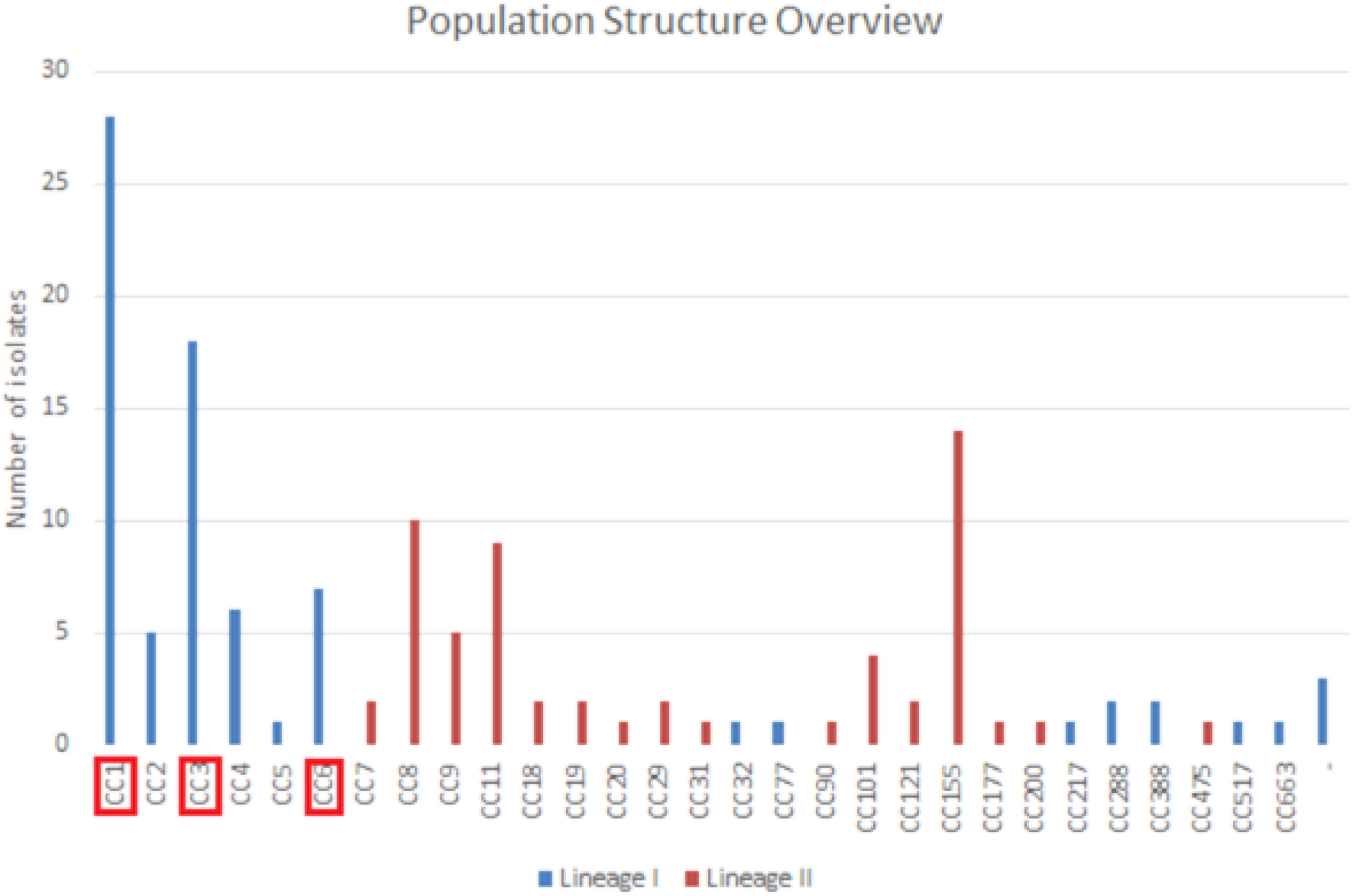
Distribution of virulence genes present in 135 *L. monocytogenes* strains.

The distribution of virulence genes was graphically represented through a heatmap (Figure 1) created in RStudio (RStudio, PBC, Boston, MA, United States).

Phylogenetic relationships among isolates were assessed using the SPREAD module within GenPat, which facilitated the analysis of cgMLST allelic profiles calculated using ChewBBACA 2.8.5 (Silva et al., 2018). A minimum spanning tree (MST) was generated with GrapeTree (Zhou et al., 2018), whereas the maximum likelihood phylogenetic tree was constructed and visualized using ETE (Huerta-Cepas et al., 2016).

## 3 Results

### 3.1 Serogroups

The 135 *L. monocytogenes* strains analyzed in this study belong to the following molecular serogroups: IVb (40%; *n* = 54), IIb (31.1%; *n* = 42), IIa (25.2%; *n* = 34), and IIc (3.7%; *n* = 5). 135 samples were taken from blood and cerebrospinal fluid in the following proportions: 118 (87.4%) from blood and 17 (12.6%) from cerebrospinal fluid.

### 3.2 Bioinformatics analysis

Detailed sequencing quality metrics, assembly statistics, contamination checks, and bioinformatic analysis parameters are provided in Supplementary Table S1. Specifically, Supplementary material includes raw-read quality metrics (total reads, Q30 scores, average read quality, and read length after trimming), assembly statistics (number of contigs, largest contig, total genome length, N50, N75, L50, and L75 values), species assignment and taxonomic classification results, sequencing coverage metrics, cgMLST quality indicators (including percentage and number of called loci).

**FIGURE 2:**
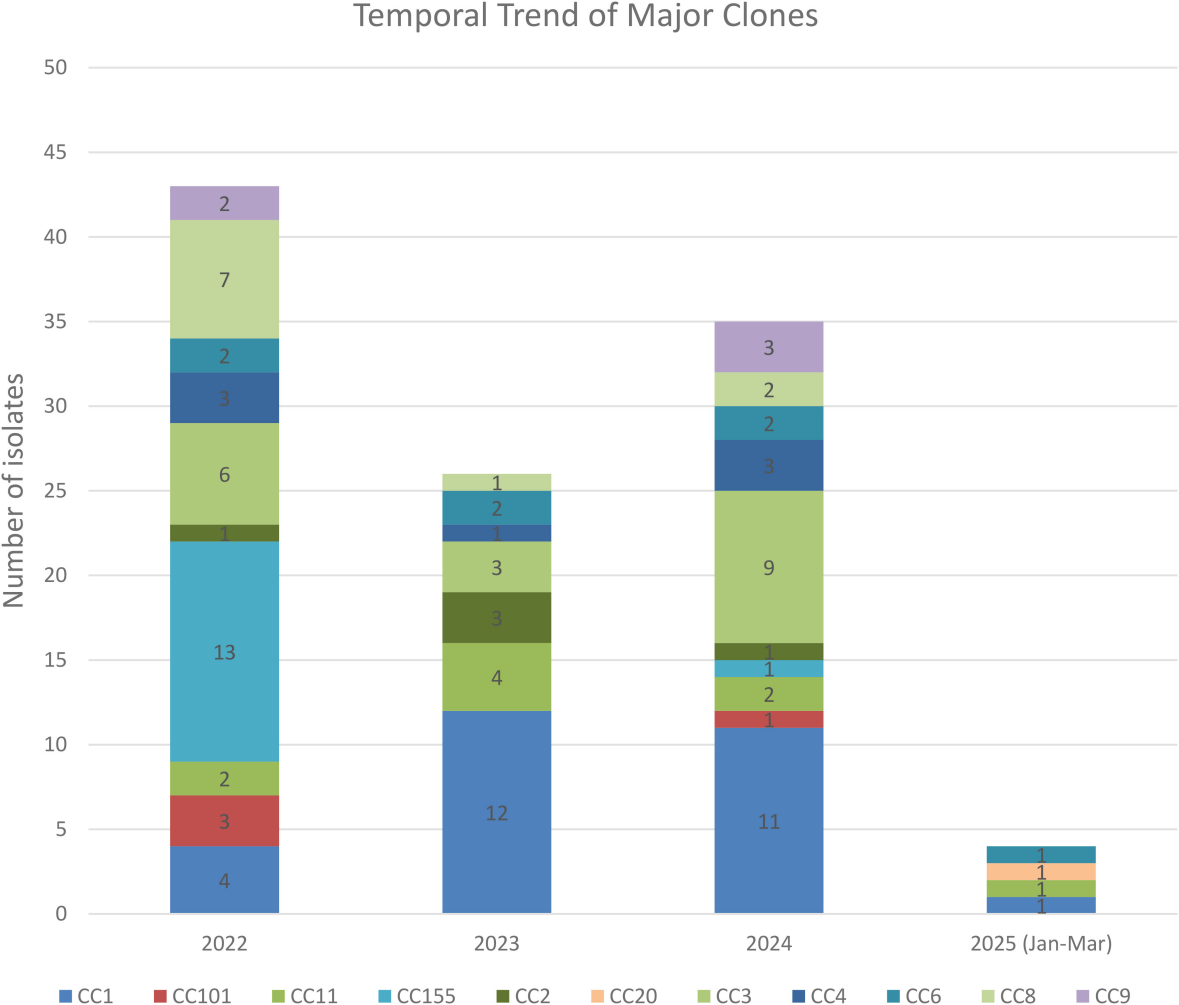
Distribution of major clonal complexes among invasive isolates. On the *x*-axis we find the lineage of belonging, while on the *y*-axis the number of isolates; the red boxes highlighting Lineage I hypervirulent clones relevant for foodborne transmission (CC1, CC3, CC6).

**FIGURE 3:**
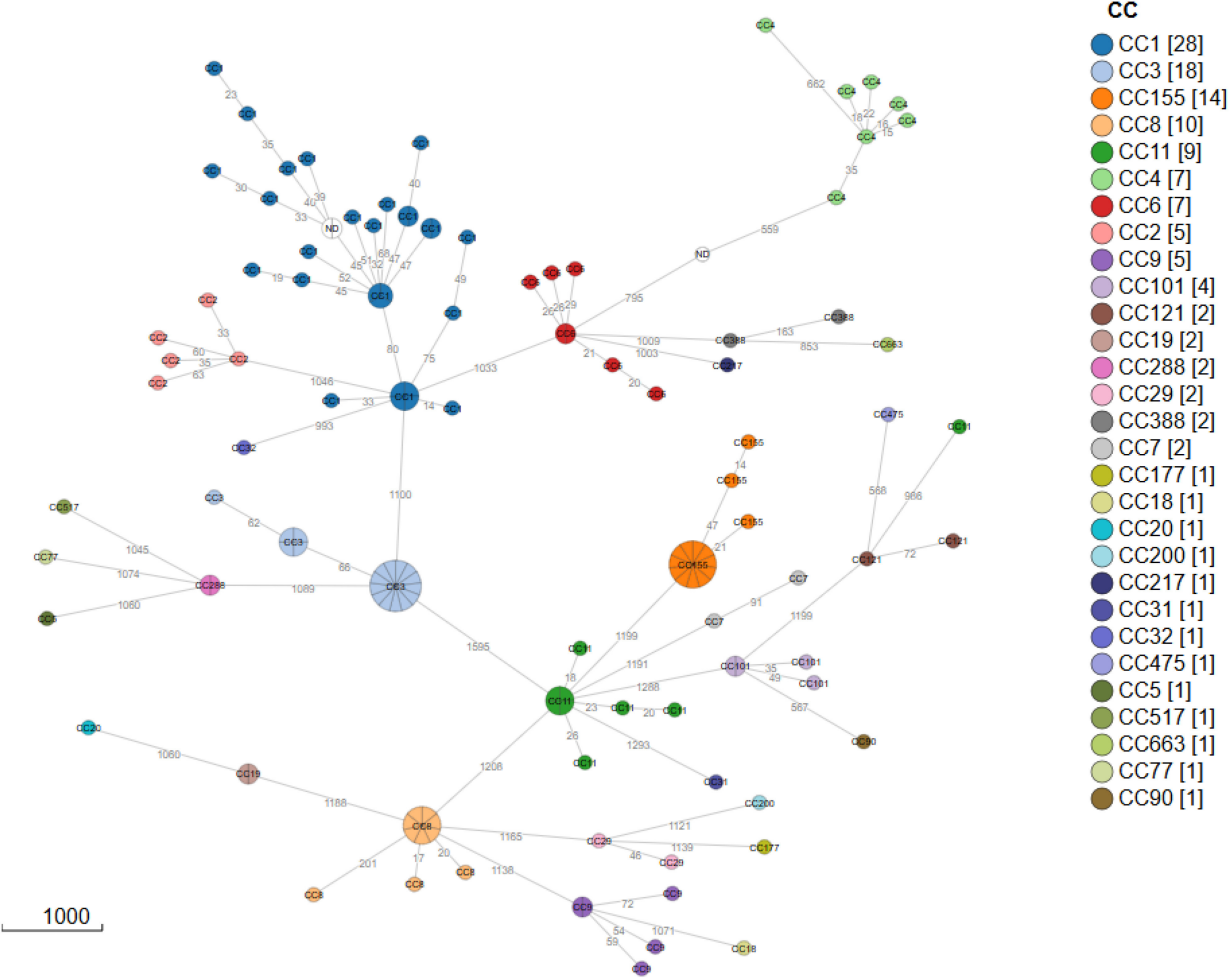
Temporal distribution of the major *L. monocytogenes* clonal complexes (CCs) identified among invasive isolates collected in Northwest Italy between 2022 and 2025.

Among the 135 *L. monocytogenes* strains analyzed, 36 distinct STs were identified: the most representative were ST1 (20%; *n* = 27), ST3 (13.3%; *n* = 18), ST155 (10.4%; *n* = 14), ST8 (6.7%; *n* = 9), ST451 (5.9%; *n* = 8) and ST6 (5.2%, *n* = 7) (Table 1). The cgMLST assigned with ChewBBACA 2.8.5 allowed to arrange the strains into 29 different clonal complexes (CCs) (Figure 2 and Table 2). Figure 3 depicts the annual frequency of major clones, over the study period (*n* ≥ 4).

**TABLE 1:** Genomic characteristics of *L. monocytogenes* strains obtained by WGS: table includes STs, CCs, serogroups distribution.

No. of isolates (%)	Sequence type	Serogroup
27 (20%)	ST1	IVb
18 (13.3%)	ST3	IIb
14 (10.4%)	ST155	IIb
9 (6.7%)	ST8	IIa
8 (5.9%)	ST451	IIa
7 (5.2%)	ST6	IVb
6 (4.4%)	ST219	IVb
5 (3.7%)	ST2	IVb
4 (3%)	ST101	IIa
4 (3%)	ST9	IIc
2 (1.5%)	ST121	IIa
2 (1.5%)	ST288	IIb
2 (1.5%)	ST29	IIa
2 (1.5%)	ST388	IVb
2 (1.5%)	ST398	IIa
2 (1.5%)	−	IVb
1 (0.7%)	ST11	IIa
1 (0.7%)	ST16	IIa
1 (0.7%)	ST177	IIa
1 (0.7%)	ST18	IIa
1 (0.7%)	ST20	IIa
1 (0.7%)	ST200	IIa
1 (0.7%)	ST217	IVb
1 (0.7%)	ST32	IVb
1 (0.7%)	ST325	IIa
1 (0.7%)	ST4	IVb
1 (0.7%)	ST425	IIa
1 (0.7%)	ST5	IIb
1 (0.7%)	ST504	IIa
1 (0.7%)	ST511	IIa
1 (0.7%)	ST595	IVb
1 (0.7%)	ST517	IIb
1 (0.7%)	ST663	IVb
1 (0.7%)	ST7	IIa
1 (0.7%)	ST77	IIb
1 (0.7%)	ST580	IIc
1 (0.7%)	−	IIb

**TABLE 2:** Genomic characteristics of *L. monocytogenes* strains obtained by WGS: table includes CCs.

No. of isolates (%)	Clonal complex
28 (20.7%)	CC1
18 (13.3%)	CC3
14 (10.4%)	CC155
10 (7.4%)	CC8
9 (6.7%)	CC11
7 (5.2%)	CC6
7 (5.2%)	CC4
5 (3.7%)	CC2
4 (3%)	CC9
4 (3%)	CC101
3 (2.2%)	−
2 (1.5%)	CC121
2 (1.5%)	CC288
2 (1.5%)	CC29
2 (1.5%)	CC388
2 (1.5%)	CC19
2 (1.5%)	CC7
1 (0.7%)	CC177
1 (0.7%)	CC18
1 (0.7%)	CC20
1 (0.7%)	CC200
1 (0.7%)	CC217
1 (0.7%)	CC32
1 (0.7%)	CC31
1 (0.7%)	CC90
1 (0.7%)	CC5
1 (0.7%)	CC475
1 (0.7%)	CC517
1 (0.7%)	CC663
1 (0.7%)	CC77

**FIGURE 4:**
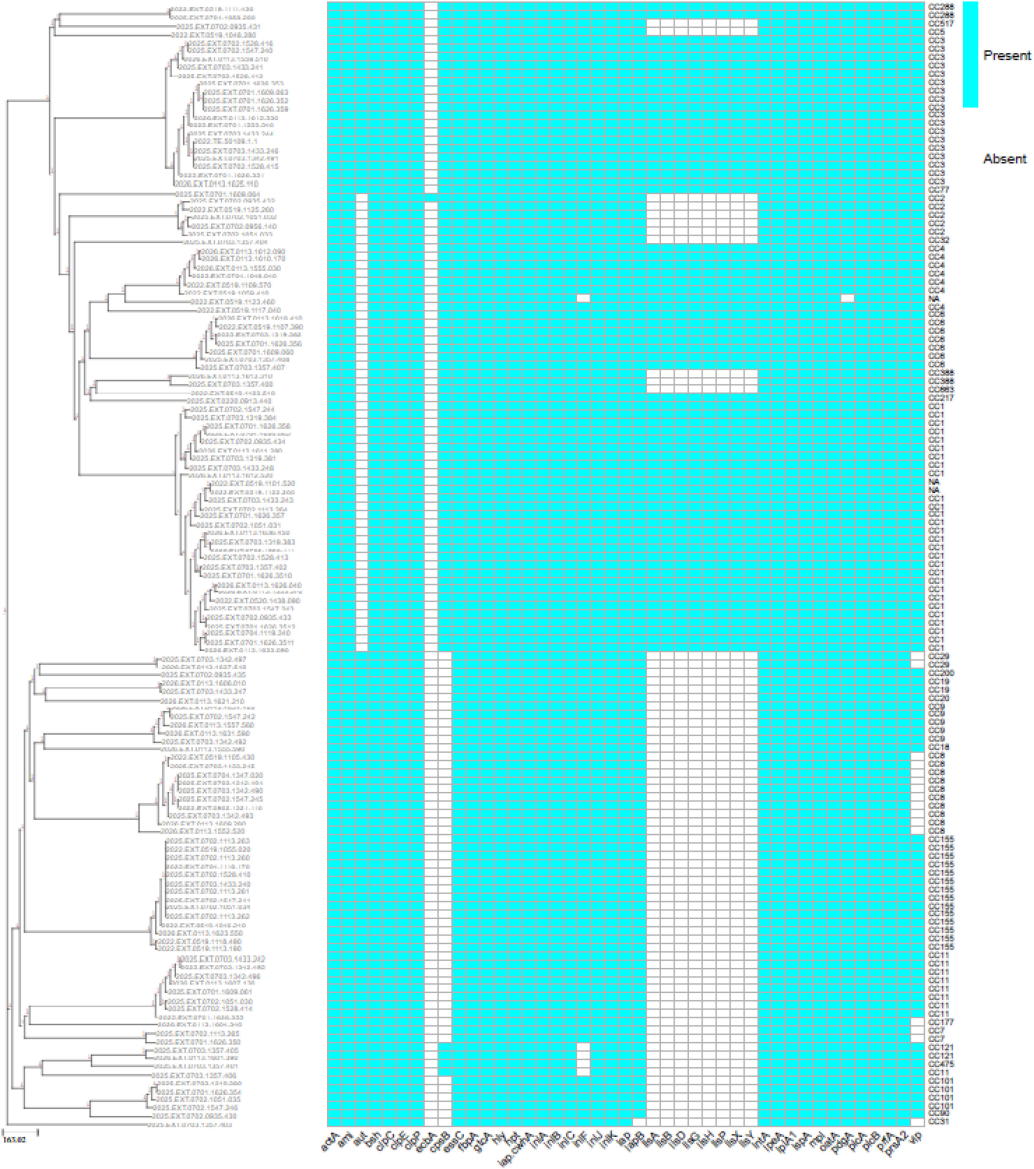
The minimum spanning tree (MST) generated with GrapeTree based on cgMLST allelic profiles generated using chewBBACA illustrates the genomic relatedness among isolates collected in Northwest Italy between 2022 and 2025. Each node represents an individual *L. monocytogenes* isolate, and edges are labeled with the number of allelic differences between genomes.

The minimum spanning tree (MST) generated with GrapeTree highlights the phylogenetic relationships between CCs, which were assessed using the SPREAD module in GenPat and the size of each CC (Figure 4).

### 3.3 Antimicrobial resistance genes

*In silico* screening of WGS data detected five resistance-associated genes: *fosX* (fosfomycin), *lmo0919 (lin)* (antibiotic ABC transporter ATPbinding protein), *nor*B (multi-drug efflux pump), *Lmon_mprF* (multiple peptide resistance factor) and *lmo0919_fam* (lincomycin resistance ABC-F-type ribosomal protection protein) in all *L. monocytogenes* strains. These genes confer resistance to fosfomycin, lincosamides, quinolones, and cationic peptides that disrupt the cell membrane such as defensins.

### 3.4 Virulence genes

In our study, LIPI-1 (*plc*B, *mpl, plc*A, *hly, act*A, *prf*A) and internalins *(inl*A, *inl*B, *inl*C, *inl*F, and *inl*J) genes were detected across all genomes. Furthermore, all strains harbored a consistent core set of virulence determinants (Figure 1), including: *bsh* (bile resistance), *clp*CEP (stress protein), *hly* (toxin-listeriolysin O precursor), *hpt* (metabolic adaptation), *iap/cwhA, inl*A, *inl*B, and *ipe*A (invasion), *lsp*A (peptidase), *mpl, p*lcA, *plc*B (exoenzyme), *oat*A, *pdg*A (immune evasion), and *prf*A (regulation).

Screening revealed a total of 43 virulence genes, encompassing major factors involved in bacterial cell wall modification (*gtc*A), adherence to host cells (*inl*J, *fbp*A, and *lap*), actin-based motility (*act*A), invasion (*inl*A and *inl*B), intracellular growth (*lpl*A1 and *prs*A2) and bile resistance (*bsh*), immune evasion (*pdg*A and *oat*A) and immune modulation (*inl*C, *inl*K, and *int*A). Notably, while all isolated carried standard pathogenicity island LIPI-1 and internalin *inl*ABCFJK markers, the LIPI-3 island (*Ils*ABDGHYXP), —classically associated with *Listeria* serotype IVb – was also identified. Specifically, within Lineage I, the CC1, CC3, CC4, and CC6 strains carried LIPI-3 (*lls*A, *lls*G, *lls*H, *lls*X, *lls*B, *lls*Y, *lls*D, and *lls*P), whereas none of the studied *L. monocytogenes* harbored a complete LIPI-4 (*lm4b_02324, lm4b_02325, lm4b_02326, lm4b_02327, lm4b_02328, lm4b_02329*) (Figure 1).

The MST generated from cgMLST allelic profiles highlighted the genomic relationships among the 135 *L. monocytogenes* isolates analyzed in this study (Figure 4). Cluster definition was performed using a threshold of ≤ 10 allelic differences, according to previously published criteria for *L. monocytogenes* genomic surveillance (Deneke et al., 2021). The analysis revealed the presence of several genetically related groups corresponding to the predominant clonal complexes identified in this study, including CC1, CC3, CC155, and CC6. However, most isolates were distributed across stable phylogenetic clusters without evidence of large, highly compact groups suggestive of acute outbreak-associated expansion during the study period.

## 4 Discussion

Due to its virulence and ability to persist and spread in the environment*, L. monocytogenes* remains a critical threat to food safety and public health. In 2024, listeriosis continued to be the foodborne infection associated with the highest rates of hospitalization and mortality in industrialized countries (EFSA) (European Food Safety Authority [EFSA], and European Centre for Disease Prevention and Control [ECDC]., 2025). To improve the characterization of foodborne pathogens, the application of WGS has significantly transformed research and routine surveillance. Currently, laboratories worldwide are rapidly expanding the WGS use to comprehensively investigate pathogens, particularly during outbreak scenarios. Consequently, WGS is increasingly integrated into national programs, outbreak investigation and environmental monitoring of food processing facilities as the primary epidemio-logical tool to support food safety and protect public health. Although its application has been more common in outbreak investigations than in routine surveillance, WGS enhances understanding of contamination sources, cross-contamination pathways, environmental reservoirs, and the long-term persistence of *L. monocytogenes*.

Recent studies reveal that *L. monocytogenes* population is largely clonal with numerous STs, and major CCs have been identified worldwide. Lineage I (associated with serotypes 1/2b, 4b), and lineage II (associated with serotype 1/2a) are responsible for > 95% of human illness (Kathariou, 2002). Furthermore, it was observed that lineage I strains tend to be more virulent and are more frequently responsible for human listeriosis outbreaks (Song et al., 2025). Overall, the clinical isolates in our study were categorized into molecular serogroups corresponding to serotypes 1/2a, 1/2b, 4b, and 1/2c. Serotypes 1/2a, 1/2b, and 4b account for the vast majority of human listeriosis cases worldwide, whereas serotype 1/2c is only rarely associated with invasive disease (Orsi et al., 2011). Serotypes 1/2a and 1/2b were likely to possess greater invasion ability than 1/2c (rarely involved) in listeriosis and may cross the placental barrier (Radoshevich and Cossart, 2018). A study reported that 95% of isolates in listeriosis were of serotypes 1/2a, 1/2b, and 4b (Althaus et al., 2014).

Our findings demonstrates that invasive listeriosis in Northwest Italy between 2022 and 2025 was largely driven by a limited number of well-established clonal complexes, particularly CC1, CC3, CC155, CC8, and CC6. These CCs are consistent with those reported in other European and international investigations of invasive listeriosis (Chenal-Francisque et al., 2011; Shinohara et al., 2025).

Notably, CC1 and CC6 are associated with the highest incidences of infection. These clones alongside CC4, are strongly linked to clinical origin (Maury et al., 2016) and harbor several pathogenic markers, highlighting a significant hazard to public health (Lachtara et al., 2021). The presence of LIPI-1 and LIPI-3 further confirms their invasive nature (Vilchis-Rangel et al., 2019). The predominance of Lineage I clones (CC1, CC3, and CC155), observed in this study aligns with previous evidence linking these clonal complexes to invasive human disease and foodborne transmission. The cgMLST-based MST analysis, applying a threshold of ≤ 10 allelic differences, further supported the hypothesis of endemic persistence of major *L. monocytogenes* clonal complexes in Northwest Italy rather than the occurrence of short-term outbreak-driven dissemination. The absence of large clusters characterized by very limited allelic variation suggests the long-term circulation of stable hypervirulent lineages within the regional food production and environmental ecosystem. The stability of these predominant clones across consecutive years suggests a possible model of endemic circulation; however, future investigations integrating clinical, food, and environmental isolates within a coordinated One Health genomic framework will be essential to confirm this hypothesis and clarify transmission pathways. These findings reinforce the concept that certain lineages exhibit enhanced ecological fitness, enabling long-term persistence in food production and processing environments while maintaining high pathogenic potential. Consequently, the recurring detection of these clones in invasive cases suggests either the presence of persistent contamination sources within the food chain or repeated reintroduction from widespread environmental or industrial reservoirs. This underscores the necessity of sustained genomic surveillance within integrated food safety systems. The distribution of the major clonal complexes observed in this study shows some differences when compared with previously published studies (Maury et al., 2016). In particular, when compared with the dataset analyzed by Maury et al. (2016) our study reported a higher proportion of CC155 (ST155) isolates, a sequence type involved in multinational clusters reported by the ECDC. The presence of this CC indicates the recent dissemination of this clone in Europe (European Centre for Disease Prevention and Control, 2023). Although this study did not focus on direct outbreak investigation, the presence of this clone in invasive cases emphasizes the importance of harmonized genomic surveillance and real-time data sharing at both national and European levels. The routine application of cgMLST-based analysis enables the early detection of expanding epidemic lineages, thereby strengthening preventive interventions before large-scale outbreaks occur.

The virulence gene profile observed in this study was consistent with the established pathogenic architecture of L. monocytogenes. All isolates carried LIPI-1, confirming its essential role in intracellular survival and systemic infection. While the presence of these markers indicates a high pathogenic potential, it should be noted that the identification of virulence genes by WGS represents genetic potential; further functional studies would be required to characterize the effective virulence level and phenotypic expression of these clones.

The resistome analysis primarily revealed intrinsic resistance determinants commonly described in *L. monocytogenes*.

Overall, our findings suggest that invasive listeriosis in Northwest Italy is sustained by the persistent circulation of a limited number of successful clonal complexes rather than by rapid genomic diversification. This clonal stability reinforces the importance of integrated surveillance strategies linking clinical isolates with food and environmental monitoring data. The routine implementation of WGS within regional public health laboratories represents a critical tool to enhance traceability, identify recurrent clones, and support risk assessment in the food production chain.

In the context of the One Health framework, genomic surveillance of clinical isolates should be considered an integral component of a comprehensive food safety system. The harmonization of genomic data across human, food, and environmental sectors will be essential to improve early detection of contamination sources, limit the spread of hyper-virulent clones, and reduce the burden of invasive listeriosis.

## 5 Conclusion

Integrated WGS based surveillance should be regarded as a core component of modern food safety systems. High-resolution genomic typing enables accurate characterization of circulating clonal complexes, supports the detection of persistent lineages, and facilitates the comparison of isolates across temporal and spatial scales. In this context, genomic data can inform evidence-based, risk-oriented control strategies aimed at reducing the burden of invasive listeriosis.

Future investigations integrating clinical, food, and environmental isolates within a coordinated One Health genomic framework will be essential to clarify transmission pathways and to distinguish endemic persistence from repeated reintroduction events. Integrative approaches of this nature have been demonstrated to enhance source attribution efforts and facilitate the development of targeted mitigation measures designed to limit the long-term circulation of hypervirulent clones within the food chain.

## Data Availability

The datasets presented in this article are not readily accessible, as the samples fall under the authority of the Ministry of Health. The data sets used and/or analyzed during the current study are available from the corresponding author upon reasonable request.
